# Mental Health Care Utilization Following Eviction Moratorium Expirations

**DOI:** 10.1001/jamahealthforum.2026.1212

**Published:** 2026-05-22

**Authors:** Yimin Ge, Kathryn M. Leifheit, Alene Kennedy-Hendricks, Iraj Qureshi, Sabriya Linton, Michael Fingerhood, Leah Robinson, Craig Evan Pollack, Matthew D. Eisenberg

**Affiliations:** 1 Department of Health Policy and Management, Johns Hopkins Bloomberg School of Public Health, Baltimore, Maryland; 2 Johns Hopkins Center for Mental Health and Addiction Policy, Baltimore, Maryland; 3 Department of Health Policy and Management, University of California, Los Angeles Fielding School of Public Health, Los Angeles; 4 Department of Mental Health, Johns Hopkins Bloomberg School of Public Health, Baltimore, Maryland; 5 Johns Hopkins School of Medicine, Baltimore, Maryland; 6 Johns Hopkins School of Nursing, Baltimore, Maryland

## Abstract

**Importance:**

Housing instability is associated with adverse mental health and increased health care use. Policies that reduce eviction risk may affect mental health–related health care utilization. Eviction moratoria enacted during the COVID-19 pandemic provide a natural experiment to examine these associations.

**Objective:**

To examine whether the lifting of eviction moratoria during the pandemic was associated with changes in mental health care utilization.

**Design, Setting, and Participants:**

A synthetic difference-in-differences approach was used, with state-week level, nationwide data of all-payer records of individuals with a mental health diagnosis or medication prescription in March to August 2020 and June to December 2021. The data were analyzed from November 2024 to November 2025. Two phases of eviction moratorium expirations were analyzed. Phase 1 included staggered expiration of state eviction moratoria from March to August 2020; phase 2 assessed the expiration of the federal eviction moratorium in August 2021, when some states maintained their own state-level eviction moratoria after the federal expiration.

**Exposures:**

State-level or federal-level eviction moratorium expirations.

**Main Outcomes and Measures:**

Weekly, state-level counts of unique patients who had (1) any outpatient mental health visit, (2) any psychotropic medication prescription, (3) outpatient visits associated with mood-related disorders, (4) outpatient visits associated with serious mental illness (SMI), and (5) suicide-related visits.

**Results:**

Of 8 963 310 individuals, 62% were female, and the mean (SD) age was 42.8 (21.5) years. Expiration of moratoria was associated with statistically significant increases in the weekly number of patients who were prescribed a psychotropic medication (0.57% in phase 1 and 1.17% in phase 2) and the number of patients who received outpatient care for SMI (3.42% in phase 1 and 3.13% in phase 2). By contrast, no measurable changes were observed in the overall patient count for mental health–related outpatient visits, outpatient visits associated with mood-related conditions, or suicide-related visits.

**Conclusions and Relevance:**

The results of this differences-in-differences study suggest that the expiration of eviction moratoria was associated with increases in the mean number of patients with psychotropic medication prescriptions and outpatient visits for SMI. These findings underscore the importance of housing for psychiatric treatments and highlight the broader health care implications of housing policy decisions.

## Key Points

**Question:**

Was the expiration of state and federal eviction moratoria associated with changes in mental health care utilization?

**Findings:**

In this synthetic difference-in-differences study of 8 963 310 individuals and all 50 states that used nationwide claims data, moratorium expirations were associated with substantial increases in the number of patients receiving psychotropic medication prescriptions and having outpatient visits for serious mental illness.

**Meaning:**

The study results suggest that lifting eviction protections was associated with heightened psychiatric treatment, particularly for individuals with serious mental illness, indicating that housing policy changes play a critical role in shaping mental health care use.

## Teaser

This synthetic difference-in-differences study examines whether the lifting of eviction moratoria during the COVID-19 pandemic was associated with changes in mental health care utilization.

## Introduction

Housing security is a key social determinant of health, especially mental health. In the form of frequent moves, evictions, crowding, and unsafe living conditions, housing insecurity can disrupt access to health care and weaken social support, as well as contribute to poorer mental health outcomes,^1,2^ including higher rates of depression, anxiety, and stress.^3,4,5,6^

The COVID-19 pandemic intensified these dynamics. Unprecedented job loss and economic disruptions exacerbated the housing crisis in the US.^7^ In response, federal, state, and local governments implemented housing policies in multiple forms, such as emergency rental assistance, eviction moratoria, and right-to-counsel protections.^7,8^ Eviction moratoria led to pauses in different stages of the eviction process, and emerging evidence has indicated that they helped reduce eviction filings and curb disease transmission.^9,10,11^ By reducing exposure to the stress of housing loss, such policies appear to have had protective associations with renters’ mental health.^12,13,14^

However, little is known about the policies’ association with the utilization of mental health services. Many of these COVID-19–era housing protections were temporary, and loss of these protections could raise distress and symptom severity, increasing demand for mental health care. At the same time, housing instability may hinder access to care as people move homes or enter shelters, lose internet access, face gaps in transportation availability, or experience other disruptions.

In this study, we aimed to understand the association between eviction moratoria and mental health care utilization using nationwide administrative claims from IQVIA. We used a natural experiment by leveraging the expiration of eviction moratoria as a policy shock. We examined how housing instability and policies designed to mitigate it may translate into observed demand for care and pressure on the health care system rather than self-reported symptoms alone. The study findings may inform tenant protection policies and crisis capacity planning among health systems and payers.

## Methods

We used a synthetic difference-in-differences approach to compare state-week use before and after eviction moratoria expired vs those where eviction moratoria remained in place. The study was divided into 2 distinct phases: when state moratoria expired (phase 1: March-August 2020) and when the federal moratorium expired (phase 2: June to December 2021). The division into 2 phases allowed us to separately evaluate the association of state-level expirations early in the pandemic and the expiration of the federal moratorium after nearly a year of nationwide protections, providing insights into whether the timing and scope of moratorium expiration was associated with outcomes. This study was deemed non–human participants research by the Johns Hopkins institutional review board and followed the Strengthening the Reporting of Observational Studies in Epidemiology (STROBE) reporting guideline.

### Data

Data to construct outcomes came from the US longitudinal prescription and nonadjudicated medical claims (Dx) files from IQVIA.^15^ Longitudinal prescription data contain about 4 billion prescription claims each year and cover roughly 70% to 90% of dispensed prescriptions across retail, mail-order, and long-term care pharmacies. The Dx data include more than 1.35 billion medical claims per year and capture services from about 70% of physicians listed by the American Medical Association. Dx records come from office-based clinicians, ambulatory centers, hospitals, skilled nursing facilities, and home health clinicians and include patient-level diagnosis codes.^15,16^

### Sample Identification

The sample of the IQVIA data consisted of patients with at least 1 mental health or substance use disorder diagnosis code between January to December from 2015 to 2022, along with their complete set of medical and pharmacy claims during the same period. To reduce bias from clinicians entering or exiting the IQVIA data collection frame, we restricted Dx claims to those submitted by clinicians with at least 1 claim per year between January and December from 2019 to 2022. The analytic dataset was organized at the state-week level from March 2020 to December 2021, which aligned with the timing of moratorium implementations and expirations. State assignment was based on the patient’s address at the time of each claim and updated if a person relocated.

### Outcome Measures

We assessed the association of eviction moratorium expiration with 5 outcomes, including the number of patients in each state-week with at least 1 claim for any (1) outpatient mental health-related visit, (2) psychotropic medication prescription, (3) mood-related outpatient visit, (4) serious mental illness (SMI) outpatient visit, and (5) suicide-related visit in any setting. The outcomes (numbers 3-5) captured increasing levels of severity, given that eviction risk might have heterogeneous effects depending on the health needs of patients. Mental health conditions were identified using *International Statistical Classification of Diseases and Related Health Problems, Tenth Revision (ICD-10)* codes in the medical claims (eTable 1 in Supplement 1), and psychotropic medication use was identified using national drug codes in the pharmacy claims (eTable 2 in Supplement 1). Outpatient services were defined based on place-of-service descriptions (eTable 3 in Supplement 1). We restricted several outcomes to outpatient to capture routine mental health treatment in a setting that is more consistently observed and comparable across states. In contrast, emergency department and inpatient claims are more heterogeneous, less comprehensively captured, and more likely to reflect short-term crisis care; therefore, focusing on outpatient visits provides a cleaner measure of treatment contact. We did not restrict suicide-related encounters to outpatient settings because these events are rare and often occur in short-term settings; limiting to outpatient would substantially reduce the sample size and statistical power.

### Exposure

A team of legal scholars reviewed legal text to define the expiration dates of state eviction moratoria.^17^ The federal expiration was defined as the date the US Supreme Court ruling ended the nationwide eviction moratorium from the US Centers for Disease Control and Prevention.

**Figure 1.**
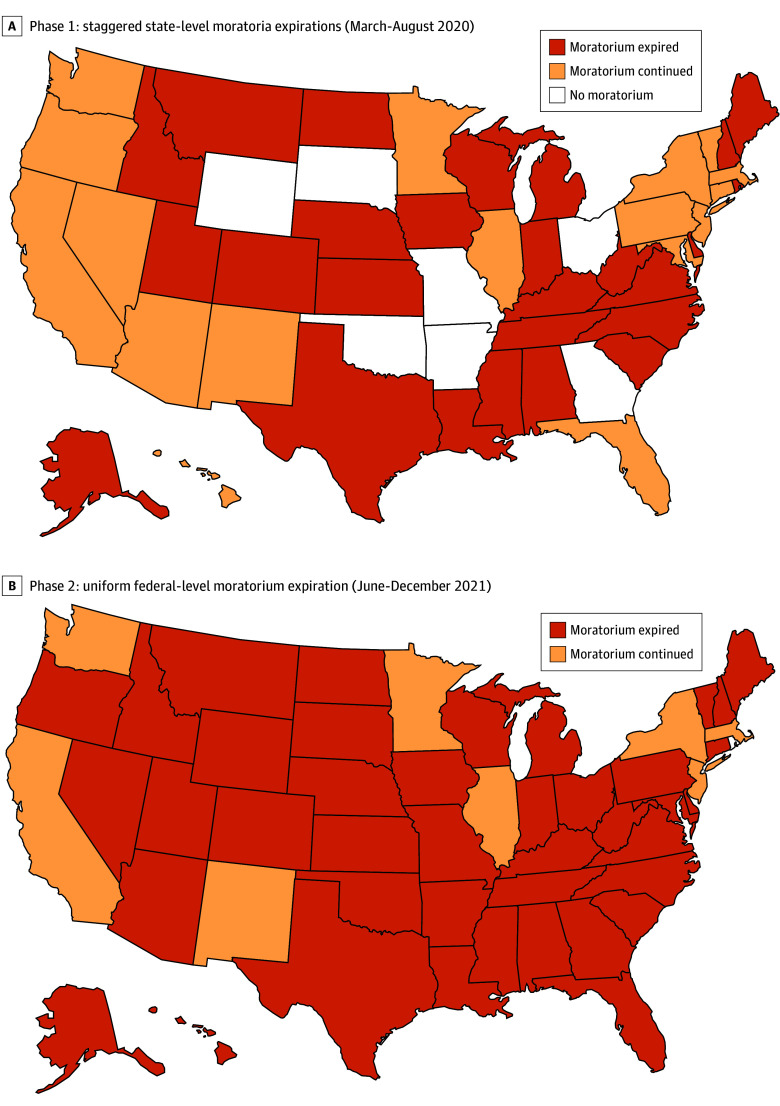
Map of Study Design and Eviction Moratorium Expirations

Phase 1 analyses covered March to August 2020, which corresponded to the early months of the COVID-19 pandemic before the federal eviction moratorium was instituted by the US Centers for Disease Control and Prevention. During this phase, 44 states implemented state-level eviction moratoria at some point in response to the federal public health emergency. Of these, 26 states (59.1%) allowed their moratoria to expire during the period, which we defined as the treatment group, and the expirations were the treatment. The remaining 18 states (40.1%) maintained state-level moratoria throughout this phase and served as the comparison group. Figure 1A shows the states in different groups and the staggered expirations of their policies.

Phase 2 analyses covered June to December 2021, which corresponded to the expiration of the federal eviction moratorium. The federal moratorium, which was enacted in September 2020, remained in effect until August 2021. Following its expiration, 9 jurisdictions, including California, Washington, DC, Illinois, Massachusetts, Minnesota, New Jersey, New Mexico, New York, and Washington, kept their state-level moratoria in place, while the remaining 42 states no longer had active state moratoria. For this phase, the treatment group was defined as the 42 states where evictions resumed after the federal moratorium expired, and the comparison group consisted of the 9 jurisdictions that retained state-level protections blocking evictions (Figure 1B).

By comparing states where protections expired with those where they remained in place, we aimed to isolate the associations of moratorium expiration from broader pandemic dynamics. The design in the second phase assumes that federal and state-level moratoria had comparable associations with housing stability and related outcomes. The validity of this assumption is supported by a Federal Reserve FEDS Note, which documented that state and federal moratoria were associated with roughly a 20% reduction compared with baseline.^18^ State moratoria were slightly stronger than federal moratoria, so we believe it is appropriate to assume that the control states were largely unaffected by federal moratorium expiration.^18^

### Covariates

We adjusted for a set of time-varying policies to account for evolving contextual factors that could plausibly influence the utilization of mental health services. Specifically, we included weekly state-level indicators for the availability of Supplemental Nutrition Assistance Program (SNAP) emergency allotments^19^ and the amount of enhanced unemployment insurance benefits,^20^ both of which directly supported household income and food security during the COVID-19 crisis. We also controlled for Medicaid expansion status,^21^ which captured broader differences in insurance coverage and access to behavioral health services across states. To account for other housing stability interventions, we included per capita state-month disbursements of emergency rental assistance funds.^22^ The program, introduced during the pandemic, provided rental support to households at risk of eviction and thus may have mitigated some of the adverse consequences of expiring moratoria by buffering the association between housing instability and mental health service demand.

### Statistical Analysis

We used a synthetic difference-in-differences (SDID) approach to address the staggered timing of state-level eviction moratorium expirations in phase 1 and better satisfy the parallel trends requirement in both phases. SDID combines synthetic control and traditional difference-in-differences (DID) by assigning optimal unit and time weights to closely match treated states’ preexposure trends.^23^ This weighting reduces dependence on the standard parallel trends assumption, preserves the invariance of DID to additive unit-level shocks, and yields more credible counterfactuals in settings with staggered adoption and heterogeneous pretrends.^24^ These optimized weights allow SDID to construct a more credible counterfactual trajectory and, in turn, estimate the average treatment effect on the treated of moratorium expirations. *Synthetic* refers to constructing a weighted combination of control states that closely reproduces the treated states’ pretreatment outcome path. SDID derives these weights from the panel data by choosing unit weights (on control states) and time weights (on pretreatment weeks) to minimize pretreatment differences in outcomes. Unlike standard DID, SDID does not treat all control units and all periods equally, and unlike matching or propensity score weighting, the weights are not chosen to balance observed covariates or treatment propensity, but rather to balance the pretreatment outcome trajectory. SDID then applies a DID-style adjustment, which helps account for remaining level differences.

In phase 1 (March to August 2020), we defined April 29, 2020, to August 31, 2020, as the observation period. This window was chosen to maximize the number of states retained while preserving the balanced panel structure required by SDID.^24^

In phase 2 (June to December 2021), we defined June 4, 2021, to December 23, 2021, as the observation period, which corresponded to the federal moratorium’s expiration and provided a similar time window to phase 1.

For estimation, we used log-transformed weekly patient counts as the outcome variables to account for skewness. Estimated coefficients were exponentiated and interpreted as percentage changes compared with the synthetic counterfactual. The standard errors were calculated using the bootstrapping procedure, which resamples units and accounts for cross-sectional dependence.^24^ While lagged pretreatment outcomes were the primary balancing source, additional covariates described in the previous section were included in the regression adjustment step to address remaining residual imbalances after synthetic weights were constructed.

We conducted 3 sensitivity analyses. First, we explored whether accounting for state population would affect the findings by modeling policy associations with population rates of mental health service utilizations (ie, patient count per capita) in place of the primary outcome (logged counts of unique patients). In the second sensitivity analysis, we reestimated the SDID models without covariates. Because SDID relies primarily on pretreatment outcome matching to achieve balance, including covariates may not always improve model fit and can introduce bias if those covariates are affected by treatment or are unevenly distributed over time.^25^ We conducted a 2-way fixed-effects analysis as the third sensitivity analysis. The 2-way fixed-effects estimates serve as a benchmark. Consistency in the direction and magnitude of key results provided validation that our findings were not affected by weighting or fluctuations. Finally, we also added a COVID-19 policy stringency measure as an additional control for phase 1 to account for the period with multiple changes. Statistical analyses were conducted using StataNow/SE, version 19.5 (StataCorp). Statistical significance was set at the 5% level.

## Results

A summary of the sample characteristics (8 963 310 individuals; 62% female; mean [SD] age, 42.8 [21.5] years) is presented in eTable 4 in Supplement 1, and the balance between treated units and synthetic units is shown in the trajectory plots presented in eFigures 1 and 2 in Supplement 1. The plots show that the treated and synthetic trajectories were generally stable before treatment and did not exhibit obvious divergent trends before treatment exposure. At the same time, because the estimated effects for the statistically significant outcomes were small in magnitude, we would not expect to see an obvious visual separation in these plots.

### Phase 1: State-Level Expirations

**Figure 2.**
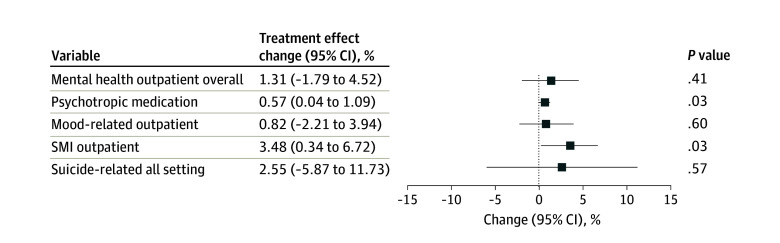
Dot Plot of the Estimated Association With Mental Health Care Use in States Where Eviction Moratorium Expired (Treated) vs States Where Eviction Moratoria Remained in Place From April 29, 2020, to August 31, 2020 SMI indicates serious mental illness.

Six treated states were excluded due to insufficient preexpiration or postexpiration data in phase 1 (eTable 5 in Supplement 1). In treated states, the mean (SD) weekly number of patients with mental health outpatient visits rose from 19 523 (17 880) to 21 107 (17 897) after expirations, compared with 43 512 (38 314) in comparison states, but no significant change was detected (1.31%; 95% CI, −1.79% to 4.52%; *P* = .41) (Table 1). The mean (SD) number of patients with psychotropic medication prescriptions declined slightly from 105 894 (95 771) to 104 843 (93 140) in treated states (157 069 [123 490] in comparison states), yet expirations were associated with a modest but significant 0.57% relative increase (95% CI, 0.04%-1.09%; *P* = .03) (Table 1). For mood-related visits, the number of patients in treated states averaged 12 297 (SD, 11 359) before and 13 105 (SD, 11 030) after expirations (26 799 [SD, 24 428] in comparison states), with no significant association (0.82%; 95% CI, −2.21%, 3.94%; *P* = .60) (Figure 2). The mean (SD) number of patients with SMI visits was stable (2616 [2219] to 2619 [2068]; 6368 [5790] in comparison states), but expirations were associated with a 3.48% increase (95% CI, 0.34%-6.72%; *P* = .03). The mean (SD) number of patients with suicide-related visits showed no meaningful change (64 [70] to 65 [68]; 139 [133] in comparison states; 2.55%; 95% CI, −5.87% to 11.73%; *P* = .57). Event-study results showing changes over time are presented in eFigure 3 in Supplement 1.

**Table 1.** Phase 1 Outcome Descriptive Statistics

Mental health visit	No. of states	Patients, mean (SD)		
		Pretreatment^a^	Posttreatment^a^	Untreated^b^
Mental health outpatient overall				
Treated states	20	19 523 (17 880)	21 107 (17 897)	NA
Untreated states	18	NA	NA	43 512 (38 314)
Psychotropic medication				
Treated states	20	105 894 (95 771)	104 843 (93 140)	NA
Untreated states	18	NA	NA	157 069 (123 490)
Mood-related outpatient				
Treated states	20	12 297 (11 359)	13 105 (11 030)	NA
Untreated states	18	NA	NA	26 799 (24 428)
SMI outpatient				
Treated states	20	2616 (2219)	2619 (2068)	NA
Untreated states	18	NA	NA	6368 (5790)
Suicide-related				
Treated states	20	64 (70)	65 (68)	NA
Untreated states	18	NA	NA	139 (133)

Abbreviations: NA, not applicable; SMI, serious mental illness.

^a^ The preperiod and postperiod means were state-week average estimates of outcome measures in the sample.

^b^ States in the comparison group included those that had state-level moratoria, but their moratorium did not expire during the observation period.

### Phase 2: Federal Expiration

**Figure 3.**
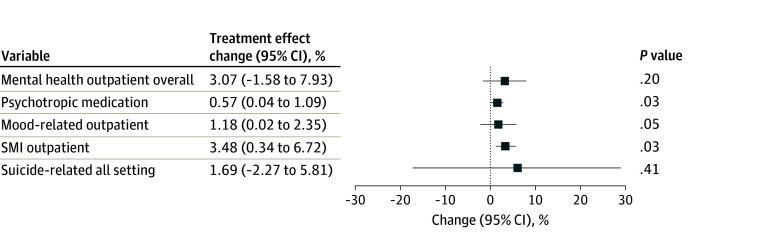
Dot Plot of the Estimated Association of the Expiration of the Federal Eviction Moratoria With Mental Health Care Use in States Without a Remaining State Moratorium (Treated) vs States With a Statewide Moratorium From June 4, 2021, to December 23, 2021 SMI, serious mental illness.

Two comparison states were excluded in phase 2 due to insufficient postexpiration data (eTable 5 in Supplement 1). In treated states, the mean (SD) weekly patient count in mental health outpatient use rose from 27 122 (23 373) to 28 001 (24 312) after the federal moratorium expired; in comparison states, this increased from 64 976 (51 219) to 65 098 (50 056) (Table 2). Consistent with phase 1, no significant change was detected (3.07%; 95% CI, −1.58% to 7.93%; *P* = .20) (Figure 3). The mean (SD) number of patients with psychotropic medication prescriptions increased from 112 880 (97 498) to 116 286 (100 270) in treated states and 201 866 (148 378) to 205 543 (150 477) in comparison states. The federal expiration was associated with a significant 1.18% increase (95% CI, 0.02%-2.35%; *P* = .047), mirroring phase 1 (Figure 3). The mean (SD) number of patients with mood-related outpatient visits rose slightly from 16 592 (14 454) to 16 872 (14 774) in treated states and declined slightly in comparison states (40 739 [32 468] to 40 681 [31 737]). No significant association was observed (1.69%; 95% CI, −2.27% to 5.81%; *P* = .41) (Figure 3). For SMI care, the mean (SD) number of patients in treated states decreased slightly (3456 [3076] to 3409 [3011]), and the number in comparison states also declined (9271 [7536] to 9012 [7239]). Nonetheless, expirations were associated with a 3.18% relative increase in SMI outpatient visits (95% CI, 1.24%-5.17%; *P* = .001), which was consistent with phase 1 (Figure 3). The mean (SD) number of patients with suicide-related visits remained stable in treated states (81 [90] to 81 [86]) and declined slightly in comparison states (175 [166] to 172 [160]). No significant change was detected (5.91%; 95% CI, −15.71% to 33.13%; *P* = .62) (Figure 3). Event-study results are shown in eFigure 4 in Supplement 1.

**Table 2.** Phase 2 Outcome Descriptive Statistics

Mental health visit	No. of states^a^	Patients, mean (SD)	
		Pretreatment^b^	Posttreatment^b^
Mental health outpatient overall			
Treated states	42	27 122 (23 373)	28 001 (24 312)
Untreated states	7	64 976 (51 219)	65 098 (50 056)
Psychotropic medication			
Treated states	42	112 880 (97 498)	116 286 (100 270)
Untreated states	7	201 866 (148 378)	205 543 (150 477)
Mood-related outpatient			
Treated states	42	16 592 (14 454)	16 872 (14 774)
Untreated states	7	40 739 (32 468)	40 681 (31 737)
SMI outpatient			
Treated states	42	3456 (3076)	3409 (3011)
Untreated states	7	9271 (7536)	9012 (7239)
Suicide-related			
Treated states	42	81 (90)	81 (86)
Untreated states	7	175 (166)	172 (161)

Abbreviation: SMI, serious mental illness.

^a^ States in the comparison group included those that continued to have state-level moratoria after the expiration of the federal moratorium.

^b^ The preperiod and postperiod means are state-week average estimates of outcome measures in the sample.

Across outcomes, 95% CI widths primarily reflected differences in sample size, event frequency, and variability. Common outcomes, such as psychotropic use, produced narrower 95% CIs, whereas rare or highly variable outcomes, like suicide-related visits, yielded wider intervals.

### Sensitivity Analysis

The results of the 2 sensitivity analyses are presented in eTable 6 in Supplement 1. Although the significance of the SMI outcome diminishes when using the per capita measure, the overall findings remained consistent with the main results.

## Discussion

In this study, we examined the association of state-level and federal-level eviction moratorium expirations on the number of people using mental health outpatient treatment overall and for specific services, including psychotropic medication, mood-related disorder outpatient care, SMI outpatient care, and suicide-related treatment. We found that the expiration of moratoria was associated with statistically significant increases in the weekly number of patients prescribed psychotropic medications and the number of patients receiving outpatient care for SMI. Our findings should be interpreted as net changes in utilization that were attributable to moratorium expiration compared with the counterfactual path, not as descriptive changes in treated states alone. By contrast, we did not see measurable changes in the number of patients with mental health–related outpatient visits overall, outpatient visits for mood-related conditions, or suicide-related encounters. Results were consistent between the 2 distinct phases, corresponding to staggered expiration of state moratoria in summer of 2020 (phase 1) and federal moratorium expiration in August 2021 (phase 2).

The results extend previous literature by demonstrating measurable increases in treatment utilization, not just mental health condition changes or subjective reports of distress,^3,4,5^ following the lifting of the eviction protections. The estimates should be interpreted as changes in observed contact with the health care system following moratorium expirations, which may reflect a combination of changes in clinical need and changes in care-seeking and access.

Assuming that worsened mental health conditions caused by housing instability^12^ lead to greater demand, one explanation of the observed increase in the population with psychotropic medication prescriptions is that pharmacological management could be a key pathway through which patients and clinicians responded to heightened psychological stressors. Compared with outpatient therapy, prescribing medication can be a relatively fast, accessible, and inexpensive intervention for patients to manage symptoms due to heightened conditions. Unlike outpatient therapy, which can be harder to access due to clinician shortages or logistical barriers, prescriptions can be maintained via telehealth or brief outpatient visits, even when navigating unstable housing. Especially during the pandemic period, when telehealth restrictions were relaxed,^26^ medication uptake may give the most immediate relief in the mental health treatment system.

The findings also suggest that the lifting of eviction protections may have disproportionately affected individuals with different psychiatric conditions. While outpatient visits for mental health overall remained relatively stable, the relative increase in the number of patients seeking treatment for SMI could suggest that housing instability may have exacerbated psychiatric symptoms among particularly vulnerable populations, potentially spurring greater demand for specialized outpatient services. Prior research has indicated that the stress associated with housing loss is more likely to precipitate clinically observable manifestations among individuals with SMI compared with those experiencing milder conditions.^27^ Individuals with SMI also face heightened risks of entering and remaining in homelessness.^28^ However, at the same time, for individuals with SMI, greater outpatient use may also reflect better ongoing management or contact with treatment, rather than worsening mental health per se, compared with other groups of patients.

From a policy perspective, these findings underscore the role of eviction moratoria as a protective public health measure during the pandemic. By preventing the immediate threat of housing loss, such policies may have buffered vulnerable populations from acute psychiatric deterioration. Conversely, their expiration appears to have increased interactions with the mental health care system, particularly in the form of medication management and outpatient SMI care. These results suggest that housing stability policies can function as upstream interventions for mental health and lapses in such protections may increase system burdens at a time when mental health services are already strained.

### Limitations

This study had several limitations. First, our analysis relied on administrative claims data, which capture treatment records of mental health, but not unmet or unrevealed needs or informal coping efforts, such as community supports. Claims data capture health care utilization, not the underlying prevalence or severity of mental health conditions. Findings should be interpreted as evidence of changes in interactions with the health care system following the removal of eviction protections rather than direct evidence of changes in underlying mental health status. We cannot disentangle whether the estimated effects reflect worsening symptoms, exacerbation of preexisting conditions, crisis-driven contact with the health care system, changes in care-seeking behavior, disruptions in treatment continuity, or increased detection and coding of mental health conditions during health care encounters. Second, our medication measure reflects only the number of patients who received at least 1 prescription, without accounting for variations in dosage strength, which may overlook important shifts in treatment intensity. Third, the observation window was relatively short, which limited our ability to detect a longer-term effect. Fourth, from March to July 2020 in phase 1, a national eviction moratorium under the CARES Act applied to properties with federally backed mortgages. Although we accounted for this period using fixed effects, these federal protections may have attenuated the observed association of state-level moratorium expirations. Fourth, we assumed a homogeneous treatment effect of the eviction moratorium and its expiration; however, in reality, the actual implementation likely varied geographically and was affected by differences in local administrative efforts.^29^ For example, some states suspended the entire eviction process, while others only halted enforcement during phase 1.^12^ In phase 2, evidence suggests that not all states fully complied with the federal moratorium, which may have affected the prepolicy period in phase 2.^30,31^ Additionally, in phase 2, some states were partially covered by their local (city or county) eviction moratoria. We tried to address this issue by consulting data on local moratoria and found that these situations were rare: there was only 1 additional case (Vermont) in which more than 10% of the population was protected by local moratoria. We excluded this state from our analysis pool, and the results remained qualitatively similar (eTable 7 in Supplement 1). Finally, the suicide outcome may not have fully captured all emergency department and inpatient treatment for suicide-related claims due to inconsistencies and limitations of the data in those settings.

## Conclusions

In this SDID study, the expiration of eviction moratoria was associated with modest increases in psychotropic medication prescriptions and outpatient treatment for SMI. These findings suggest that housing policy changes can translate into measurable changes in mental health care utilization and potentially demand on outpatient psychiatric care. The findings underscore housing stability as a potentially important upstream lever for shaping mental health care use and highlight the broader health system implications of housing policy decisions.

## Notes

Supplement 1. eTable 1. Mental Health Conditions Diagnosis Codes eTable 2. Psychotropic Medications with National Drug Codes Sample eTable 3. Places of Service Description of Outpatient eTable 4. Sample Characteristics eTable 5. Rationale for Exclusion of States from Analyses eTable 6. Sensitivity Analysis Results eTable 7. Dropping One Additional State (VT) for the Local Moratorium in Phase 2 eFigure 1. Trajectory Balance Plots Phase 1 eFigure 2. Trajectory Balance Plots Phase 2 eFigure 3. Event Study Plot Phase 1 eFigure 4. Event Study Plot Phase 2

Supplement 2. Data sharing statement
