# Supplementary material for: Mental Health Care Utilization Following Eviction Moratorium Expirations
Source: JAMA Health Forum. 2026 May 22;7(5):e261212. doi: 10.1001/jamahealthforum.2026.1212 (PMC13197875; doi:10.1001/jamahealthforum.2026.1212)
Supplement: Supplement 1. — eTable 1. Mental Health Conditions Diagnosis Codes eTable 2. Psychotropic Medications with National Drug Codes Sample eTable 3. Places of Service Description of Outpatient eTable 4. Sample Characteristics eTable 5. Rationale for Exclusion of States from Analyses eTable 6. Sensitivity Analysis Results eTable 7. Dropping One Additional State (VT) for the Local Moratorium in Phase 2 eFigure 1. Trajectory Balance Plots Phase 1 eFigure 2. Trajectory Balance Plots Phase 2 eFigure 3. Event Study Plot Phase 1 eFigure 4. Event Study Plot Phase 2 [file jamahealthforum-e261212-s001.pdf]

Supplemental Online Content

Ge Y, Leifheit KM, Kennedy-Hendricks A, et al. Mental health care utilization following eviction moratorium expirations. *JAMA Health Forum*. 2026;7(5):e261212. doi:10.1001/jamahealthforum.2026.1212

eTable 1. Mental Health Conditions Diagnosis Codes
eTable 2. Psychotropic Medications with National Drug Codes Sample
eTable 3. Places of Service Description of Outpatient
eTable 4. Sample Characteristics
eTable 5. Rationale for Exclusion of States from Analyses
eTable 6. Sensitivity Analysis Results
eTable 7. Dropping One Additional State (VT) for the Local Moratorium in Phase 2
eFigure 1. Trajectory Balance Plots Phase 1
eFigure 2. Trajectory Balance Plots Phase 2
eFigure 3. Event Study Plot Phase 1
eFigure 4. Event Study Plot Phase 2

This supplemental material has been provided by the authors to give readers additional information about their work.

© 2026 Ge Y et al. *JAMA Health Forum*.

eTable 1. Mental Health Conditions Diagnosis Codes

ICD-10 Code	Description	Sub-groups
**General**		
F20	Schizophrenia	Serious Mental Illness (SMI)
F21	Schizotypal disorder	SMI
F22	Delusional disorders	SMI
F23	Brief psychotic disorder	SMI
F24	Shared psychotic disorder	SMI
F25	Schizoaffective disorders	SMI
F28	Other psychotic disorder not due to a substance or known physiological condition	SMI
F29	Unspecified psychosis not due to a substance or known physiological condition	SMI
F30	Manic episode	SMI
F31	Bipolar disorder	SMI
F32	Depressive episode	Mood-related disorder
F33	Major depressive disorder, recurrent	Mood-related disorder
F34	Persistent mood [affective] disorders	Mood-related disorder
F39	Unspecified mood [affective] disorder	Mood-related disorder
F40	Phobic anxiety disorders	
F41	Other anxiety disorders	Mood-related disorder
F42	Obsessive-compulsive disorder	SMI
F43	Reaction to severe stress, and adjustment disorders	
F44	Dissociative and conversion disorders	
F45	Somatoform disorders	
F48	Other nonpsychotic mental disorders	
F50	Eating disorders	
F53	Mental and behavioral disorders associated with the puerperium, not elsewhere classified	
F54	Psychological and behavioral factors associated with disorders or diseases classified elsewhere	
F59	Unspecified behavioral syndromes associated with physiological disturbances and physical factors	
F60	Specific personality disorders	SMI - F60.3 personality disorder (borderline)
F63	Impulse disorders	

© 2026 Ge Y et al. *JAMA Health Forum*.

F68	Other disorders of adult personality and behavior	
F69	Unspecified disorder of adult personality and behavior	
F84	Pervasive developmental disorders	
F90	Attention-deficit hyperactivity disorders	
F91	Conduct disorders	
F93	Emotional disorders with onset specific to childhood	
F94	Disorders of social functioning with onset specific to childhood and adolescence	
F95	Tic disorder	
F98	Other behavioral and emotional disorders with onset usually occurring in childhood and adolescence	
F99	Mental disorder, not otherwise specified	
**Suicide**		
R45.851	Suicide ideation	Suicide-related
X71-X83	Intentional self-harm due to drowning and submersion, firearms, explosive or thermal material, sharp or blunt objects, jumping from a high place, jumping or lying in front of a moving object, crashing of motor vehicle, and other specified means	Suicide-related
T36-T50 with the 6th character of 2 (except for T36.9, T37.9, T39.9, T41.4, T42.7, T43.9, T45.9, T47.9, and T49.9, which are included if the 5th character is 2)	Intentional self-harm due to drug poisoning (overdose)	Suicide-related
T51-T65 with the 6th character of 2 (except for T51.9, T52.9, T53.9, T54.9, T56.9, T57.9, T58.0, T58.1, T58.9, T59.9, T60.9, T61.0, T61.1, T61.9, T62.9, T63.9, T64.0, T64.8, and T65.9, which are included if the 5th character is 2)	Intentional self-harm due to toxic effects of nonmedicinal substances	Suicide-related
T71 with the 6th character of 2	Intentional self-harm due to asphyxiation, suffocation, strangulation	Suicide-related
T14.91	Suicide attempt, unknown mechanism	Suicide-related

© 2026 Ge Y et al. *JAMA Health Forum*.

eTable 2. Psychotropic Medications with National Drug Codes Sample

Medication Class	Active Ingredients	National Drug Codes (examples)
Antidepressant	amitriptyline, amitriptyline/chlordiazepoxide, amitriptyline/perphenazine, amoxapine, brexanolone, bupropion, citalopram, clomipramine, desipramine, desvenlafaxine, doxepin, duloxetine, escitalopram, esketamine, fluoxetine, fluoxetine/olanzapine, fluvoxamine, gepirone, imipramine, isocarboxazid, levomilnacipran, maprotiline, milnacipran, mirtazapine, nefazodone, nomifensine, nortriptyline, paroxetine, perphenazine/amitriptylin, phenelzine, protriptyline, selegiline, sertraline, tranylcypromine, trazodone, trimipramine, venlafaxine, vilazodone, vortioxetine, zuranolone	50268004011, 00832865104, 58016073207, 00179184370, 42549067290, 00143980901, 16571075601, 53489037401, 42254023560, 16252053530
Anticonvulsants	brivaracetam, carbamazepine, divalproex, gabapentin, lamotrigine, levetiracetam, oxcarbazepine, pregabalin, tiagabine, topiramate, valproate, zonisamide	50474067066, 00781598701, 54569265500, 61392002965, 00440556230, 53746010210, 00173063306, 00179166171, 16590023890, 55045307601
Antipsychotic, 1st generation	chlorpromazine, chlorprothixene, fluphenazine, haloperidol, loxapine, mesoridazine, molindone, perphenazine, pimozide, prochlorperazine, promazine, thioridazine, thiothixene, trifluoperazine	17236012810, 00349204122, 58016083710, 00779047925, 00144045551, 62584072401, 00056007670, 49884054901, 00591554210, 53978209009
Antipsychotic, 2nd generation	aripiprazole, asenapine, brexpiprazole, cariprazine, clozapine, iloperidone, lumateperone, lurasidone, olanzapine, olanzapine/samidorphan, paliperidone, pimavanserin, quetiapine, risperidone, ziprasidone	58016094018, 58016094077, 00093576810, 63629337901, 50090334201, 24236017402, 52343000460, 65862012053, 49856258800, 49856258806
Benzodiazepines	alprazolam, chlordiazepoxide, clonazepam, clorazepate, diazepam, estazolam, flurazepam, halazepam, lorazepam, midazolam, oxazepam, prazepam, quazepam, remimazolam, temazepam, triazolam	54569095402, 00009372004, 24236040802, 63304055330, 52555015805, 33358010220, 00228205350, 65243018403, 00364079401, 18837028160
Injectable antipsychotic	aripiprazole, chlordiazepoxide, chlorpromazine, fluphenazine, haloperidol, loxapine, olanzapine, paliperidone, prochlorperazine, risperidone, ziprasidone	59148007280, 65757040303, 70710146301, 50458030711, 55150036025, 00000858056, 70710146301, 47202417001, 55390041205, 63323046951
Lithium	Lithium	62332001491, 00032751610, 51655024990, 00247017909, 58016047927, 68084064011, 51432025703, 50090605200, 00083656570, 00536395605
Stimulants	amphetamine, amphetamine/dextroamphetamine, dexmethylphenidate, dextroamphetamine,	00555077610, 53489056401, 54868619201, 35356014830, 70010003301, 70010003401,

© 2026 Ge Y et al. *JAMA Health Forum*.

	lisdexamfetamine, methamphetamine, methylphenidate, serdexmethylphenidate/dexmethylphenidate	72162204806, 00093504501, 68084094395, 61314036705
Other types of anti-anxiety drugs	buspirone, hydroxyzine, meprobamate, meprobamate/aspirin, meprobamate/benactyzine, meprobamate/ethoheptazine, meprobamate/tridihexethyl	58016072082, 62540566409, 51079053410, 58016025993, 51203015501, 00069541073, 52446027921, 00814472014, 52273021205, 47202221002
Other types of drugs for attention-deficit/hyperactivity disorder (ADHD)	armodafinil, atomoxetine, clonidine, guanfacine, modafinil, viloxazine	63285003201, 60687056721, 54124076930, 43063069090, 00254254328, 38779056111, 53633011110, 55812015304, 58016050503, 43353083130
Other types of drugs for hypnotic conditions	daridorexant, eszopiclone, lemborexant, ramelteon, suvorexant, zaleplon, zolpidem	58016098181, 76420002490, 52959085200, 00378527201, 49999003720, 71610027228, 16590052728, 54569576001, 58016004890, 00832125011

© 2026 Ge Y et al. *JAMA Health Forum*.

eTable 3. Places of Service Description of Outpatient

Type	Place of Service Description
Outpatient	office
	hospital outpatient
	non-residential substance abuse treatment facility
	clinic - freestanding
	on campus - outpatient hospital
	community mental health center
	independent clinic
	clinic - community mental health center
	special facility - other
	federally qualified health center
	off campus - outpatient hospital
	clinic - other
	clinic - federally qualified health center (FQHC)
	psychiatric facility - partial hospitalization
	public health clinic
	clinic - comprehensive outpatient rehabilitation facility (CORF)
	clinic - outpatient rehabilitation facility (ORF)
	rural health clinic
	comprehensive outpatient rehabilitation facility
	clinic - rural health
	school
	mobile unit
	tribal 638 free-standing facility
	Indian health service free-standing facility

© 2026 Ge Y et al. *JAMA Health Forum*.

eTable 4. Sample Characteristics

	N of unique patients	Percentage of female	Mean age (SD)
Phase 1			
Mental health outpatient overall	8,963,310	61.98%	42.83 (21.47)
Psychotropic medication	25,970,878	63.62%	49.84 (20.30)
Mood-related outpatient	6,268,058	67.70%	47.07 (20.18)
SMI outpatient	1,091,871	57.36%	45.22 (17.03)
Suicide-related all setting	54,374	60.30%	36.82 (19.34)
Phase 2			
Mental health outpatient overall	16,242,285	62.49%	41.78 (21.88)
Psychotropic medication	36,519,153	63.71%	49.02 (20.72)
Mood-related outpatient	11,543,371	68.18%	46.01 (20.63)
SMI outpatient	1,778,791	58.65%	44.55 (17.44)
Suicide-related all setting	103,760	63.13%	33.94 (19.25)

© 2026 Ge Y et al. *JAMA Health Forum*.

eTable 5. Rationale for Exclusion of States from Analyses

Dropped States	Reasons for Exclusion
**Phase 1**	
Indiana (IN)	Their state moratorium expirations happened very late in the observation window, leaving only minimal post-treatment weeks for analysis.
Kentucky (KY)	
Maine (ME)	
North Dakota (ND)	Its state moratorium expired right at the start of the analytic window.
Virginia (VA)	It reinstated a second moratorium early in our study window, which truncated its effective post-treatment period.
Kansas (KS)	It also reinstated a second moratorium during our study window.
**Phase 2**	
Illinois (IL)	Their state moratoria expirations occurred too early in the study window so that some of the post-treatment periods were truncated and failed to satisfy the balanced panel requirement of the SDID model framework.
Washington (WA)	

Notes: The six states in Phase 1 belong to the treatment group. The two states in Phase 2 belong to the comparison group.

© 2026 Ge Y et al. *JAMA Health Forum*.

eTable 6. Sensitivity Analyses Results

Outcomes	Phase 1		Phase 2	
	Percent Change (95% CI)	P-value	Percent Change (95% CI)	P-value
**Sensitivity Test 1: using count per capita as the outcome**				
Mental health outpatient overall	2.33% (-2.33%, 6.99%)	0.348	3.36% (-2.80%, 9.53%)	0.285
Psychotropic medication	0.58% (0.13%, 1.06%)	0.012	1.58% (0.56%, 2.60%)	0.002
Mood-related outpatient	1.87% (-2.61%, 6.35%)	0.460	2.69% (-3.34%, 7.21%)	0.472
SMI outpatient	3.17% (-1.59%, 7.93%)	0.123	3.27% (-0.12%, 6.66%)	0.059
Suicide-related	0.98% (-8.07%, 10.11%)	0.828	3.32% (-18.87%, 25.44%)	0.770
**Sensitivity Test 2: dropping all the covariates**				
Mental health outpatient overall	1.33% (-1.78%, 4.53%)	0.407	1.61% (-3.81%, 7.30%)	0.569
Psychotropic medication	0.57% (0.04%, 1.09%)	0.035	1.26% (0.44%, 2.07%)	0.002
Mood-related outpatient	0.82% (-2.21%, 3.94%)	0.599	0.98% (-3.00%, 5.12%)	0.634
SMI outpatient	3.48% (0.34%, 6.72%)	0.029	3.08% (-0.02%, 6.29%)	0.052
Suicide-related	2.55% (-5.86%, 11.73%)	0.564	5.19% (-4.02%, 15.28%)	0.279
**Sensitivity Test 3: two-way fixed effects analysis**				
Overall mental health outpatient	3.56% (2.12%, 5.02%)	<0.001	0.34% (-0.84% to 1.55%)	0.572
Psychotropic medication	0.35% (-0.05%, 0.76%)	0.090	0.82% (0.46% to 1.18%)	<0.001
Mood-related outpatient	2.68% (1.29%, 4.09%)	0.000	-0.03% (-1.10% to 1.05%)	0.955
SMI outpatient	2.16% (0.35%, 4.00%)	0.019	1.58% (0.22% to 2.96%)	0.022
Suicide-related all setting	5.85% (0.20%,11.82%)	0.042	5.54% (0.04% to 11.34%)	0.048
**Sensitivity Test 4: adding COVID-19 policy stringency measure as an additional covariate in Phase 1**				
Overall mental health outpatient	1.24% (-2.21%, 4.81%)	0.487		
Psychotropic medication	0.58% (0.02%, 1.14%)	0.042		

© 2026 Ge Y et al. *JAMA Health Forum*.

Mood-related outpatient	0.60% (-2.72%, 4.04%)	0.726	
SMI outpatient	3.23% (-0.12%, 6.70%)	0.059	
Suicide-related all setting	3.99% (-4.85%, 13.66%)	0.388	

© 2026 Ge Y et al. *JAMA Health Forum*.

eTable 7. Dropping One Additional State (VT) for the Local Moratorium in Phase 2

**Outcomes**	**Percent Change (95% CI)**	**P-value**
Mental health outpatient overall	2.95% (-1.38%, 7.47%)	0.185
Psychotropic medication	1.20% (0.37%, 2.03%)	0.004
Mood-related outpatient	1.57% (-1.99%, 5.36%)	0.393
SMI outpatient	2.87% (0.57%, 5.22%)	0.014
Suicide-related	4.29% (-2.64%, 11.71%)	0.231

Notes: WA and VT had local moratoria covering more than 10% of the state population. Since WA had already been dropped for the reason in Appendix Table 4, only one additional case (VT) was removed here for the test.

© 2026 Ge Y et al. *JAMA Health Forum*.

eFigure 1. Trajectory Balance Plots Phase 1

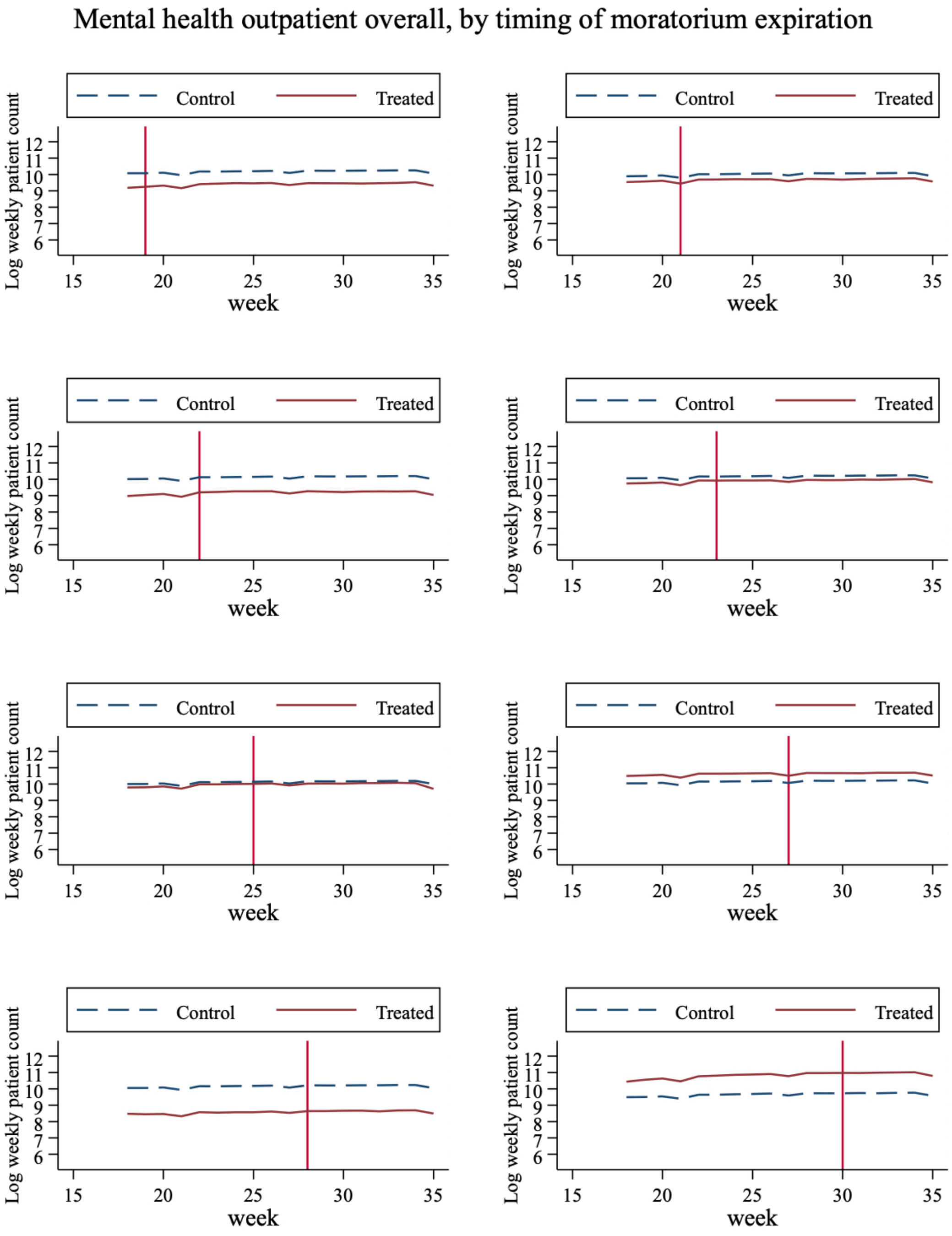

Notes: The trajectory plots are for each treatment timing group. The treated and synthetic trajectories are generally stable prior to treatment and do not exhibit obvious divergent trends before treatment exposure. Because the estimated effects for our statistically significant outcomes are small, we would not expect to see a clear visual separation in these plots.

© 2026 Ge Y et al. *JAMA Health Forum*.

Psychotropic medication, by timing of moratorium expiration

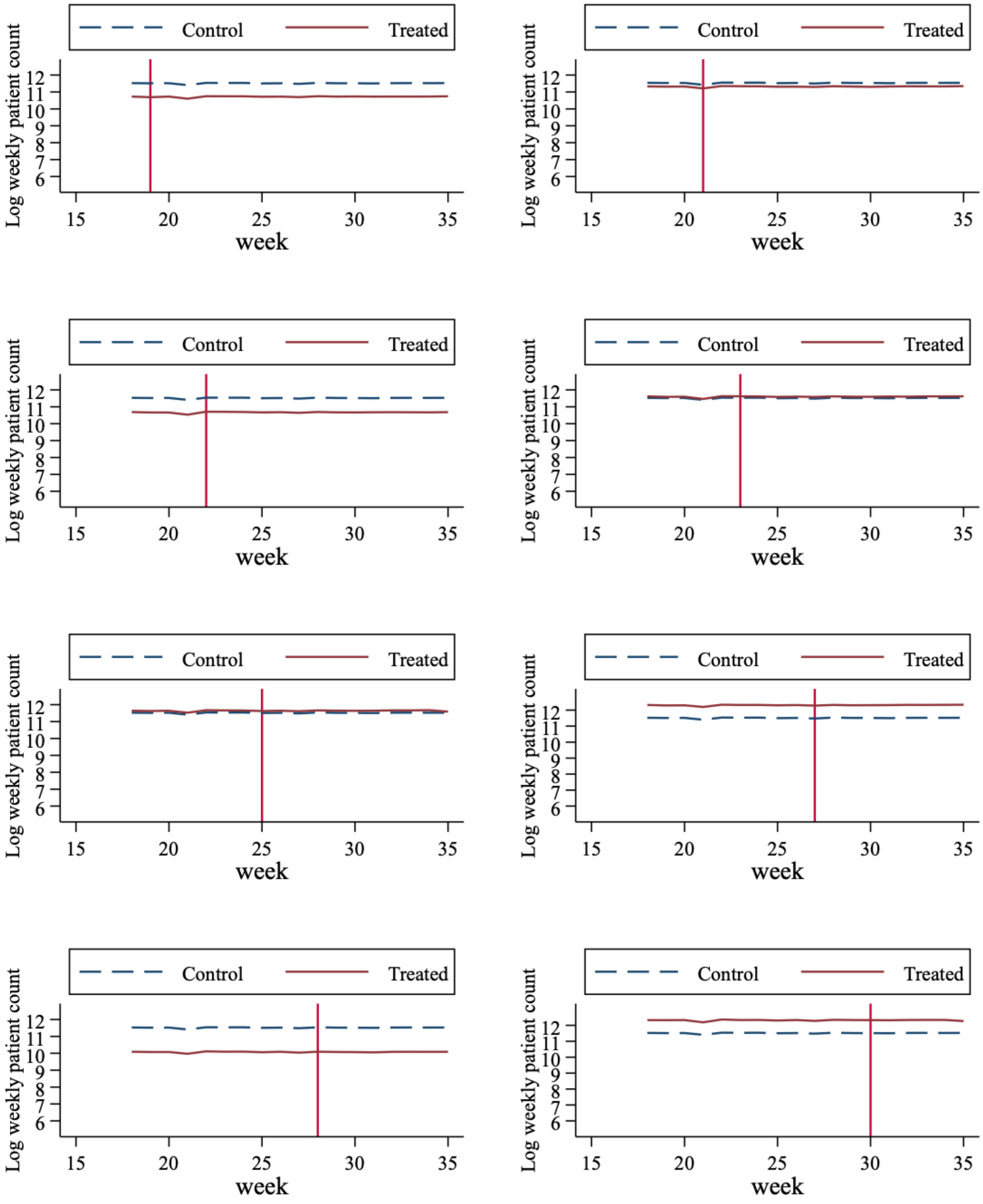

Notes: The trajectory plots are for each treatment timing group. The treated and synthetic trajectories are generally stable prior to treatment and do not exhibit obvious divergent trends before treatment exposure. Because the estimated effects for our statistically significant outcomes are small, we would not expect to see a clear visual separation in these plots.

© 2026 Ge Y et al. *JAMA Health Forum*.

Mood-related outpatient, by timing of moratorium expiration

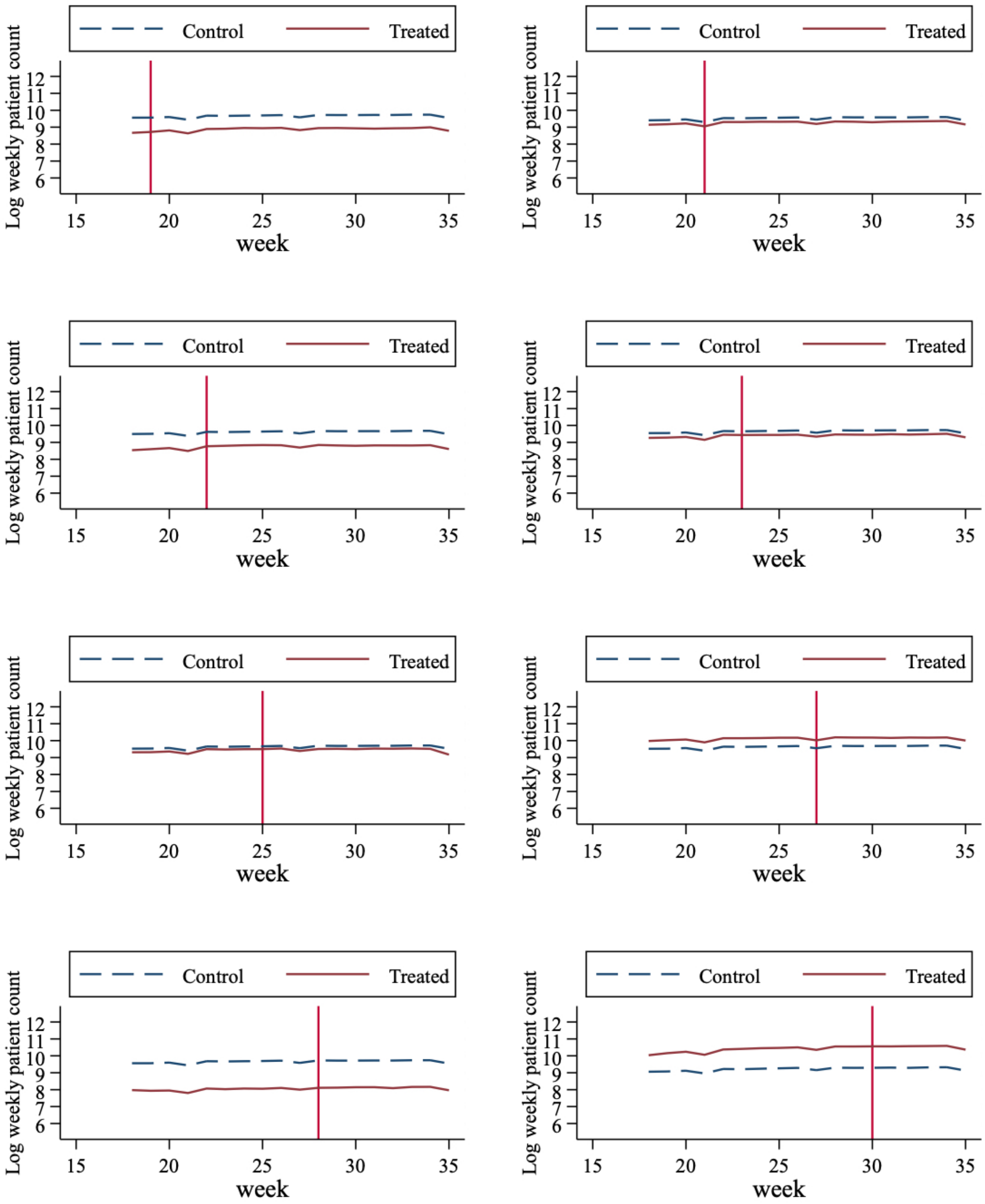

Notes: The trajectory plots are for each treatment timing group. The treated and synthetic trajectories are generally stable prior to treatment and do not exhibit obvious divergent trends before treatment exposure. Because the estimated effects for our statistically significant outcomes are small, we would not expect to see a clear visual separation in these plots.

© 2026 Ge Y et al. *JAMA Health Forum*.

SMI outpatient, by timing of moratorium expiration

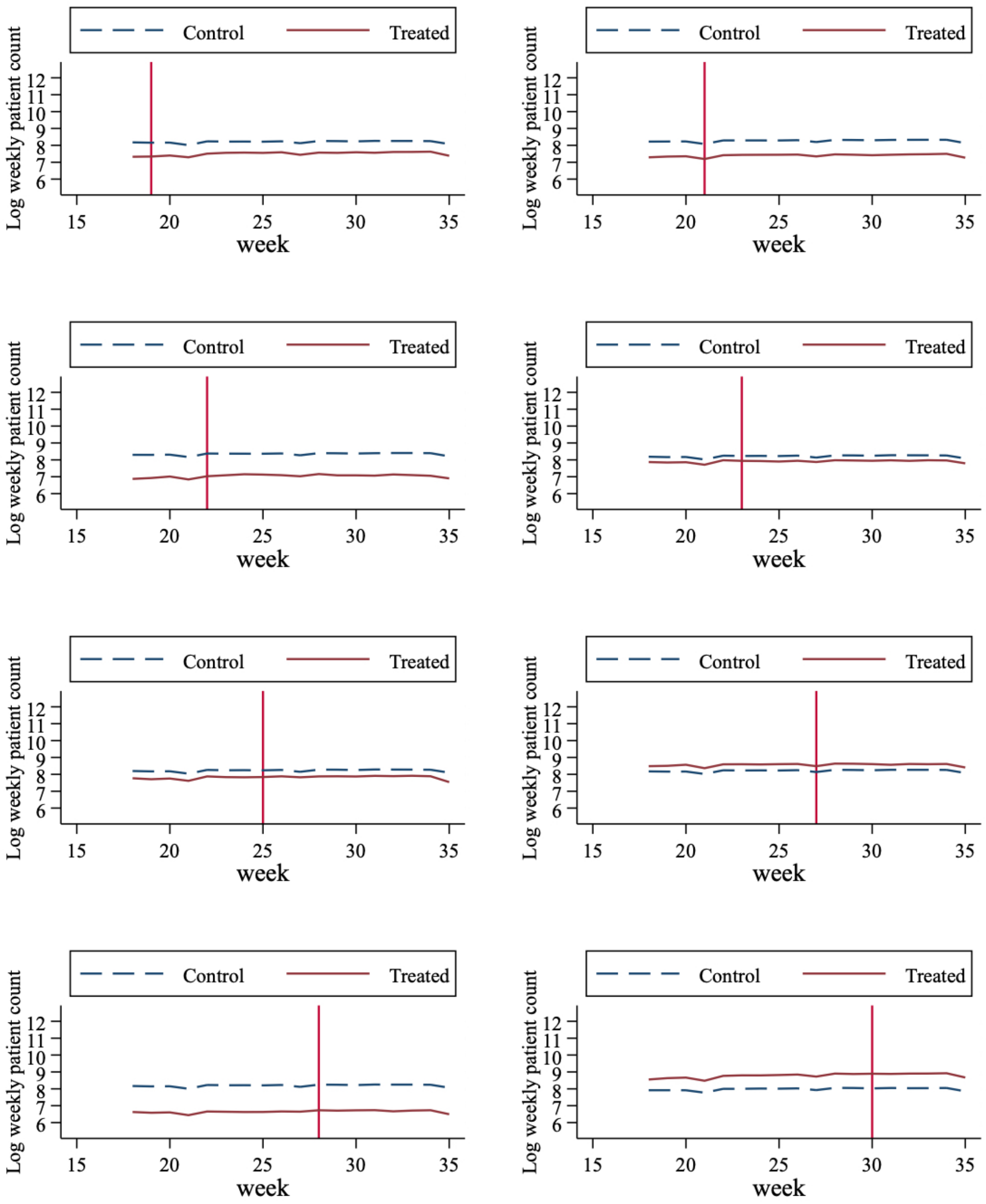

Notes: The trajectory plots are for each treatment timing group. The treated and synthetic trajectories are generally stable prior to treatment and do not exhibit obvious divergent trends before treatment exposure. Because the estimated effects for our statistically significant outcomes are small, we would not expect to see a clear visual separation in these plots.

© 2026 Ge Y et al. *JAMA Health Forum*.

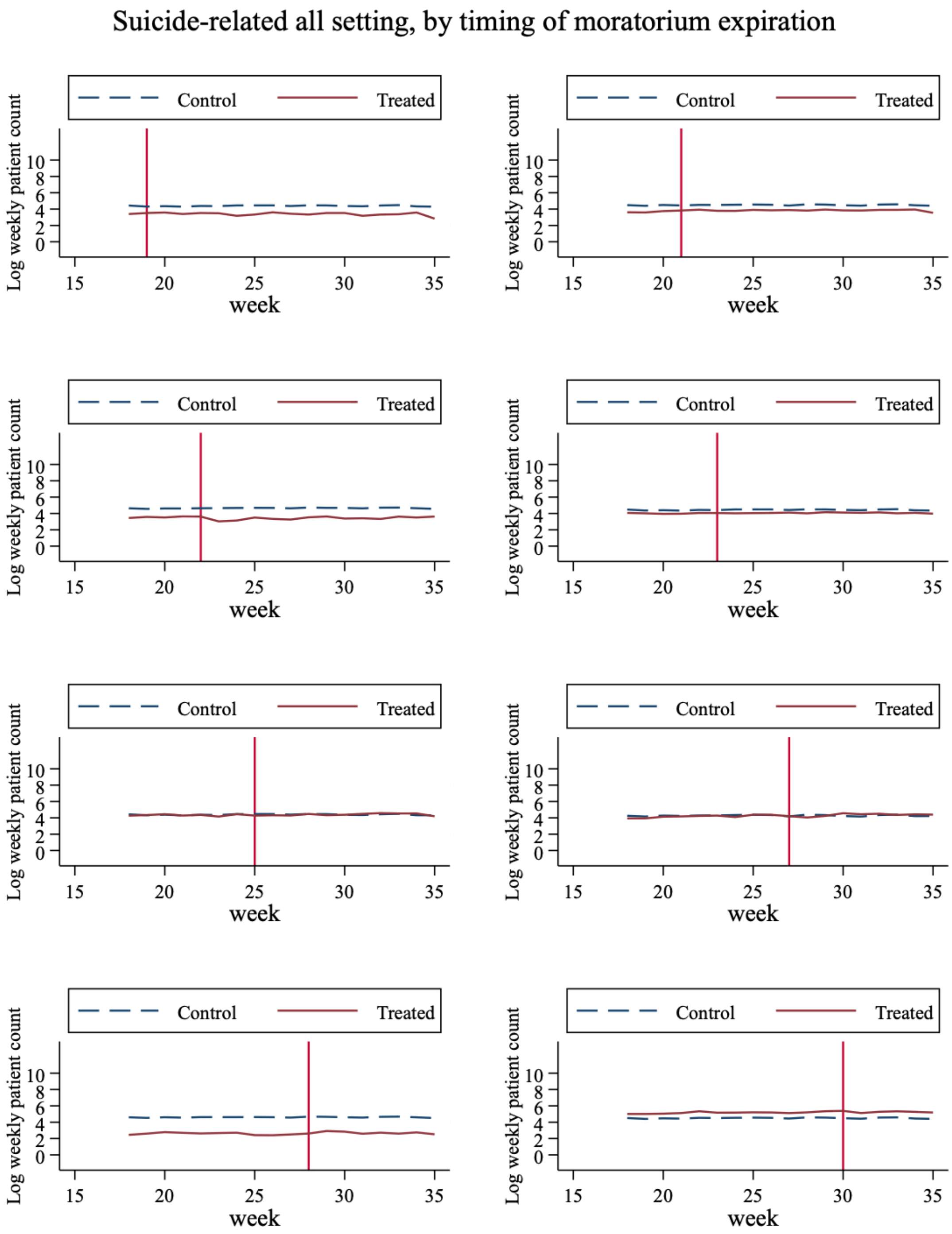

Notes: The trajectory plots are for each treatment timing group. The treated and synthetic trajectories are generally stable prior to treatment and do not exhibit obvious divergent trends before treatment exposure. Because the estimated effects for our statistically significant outcomes are small, we would not expect to see a clear visual separation in these plots.

© 2026 Ge Y et al. *JAMA Health Forum*.

eFigure 2. Trajectory Balance Plots Phase 2

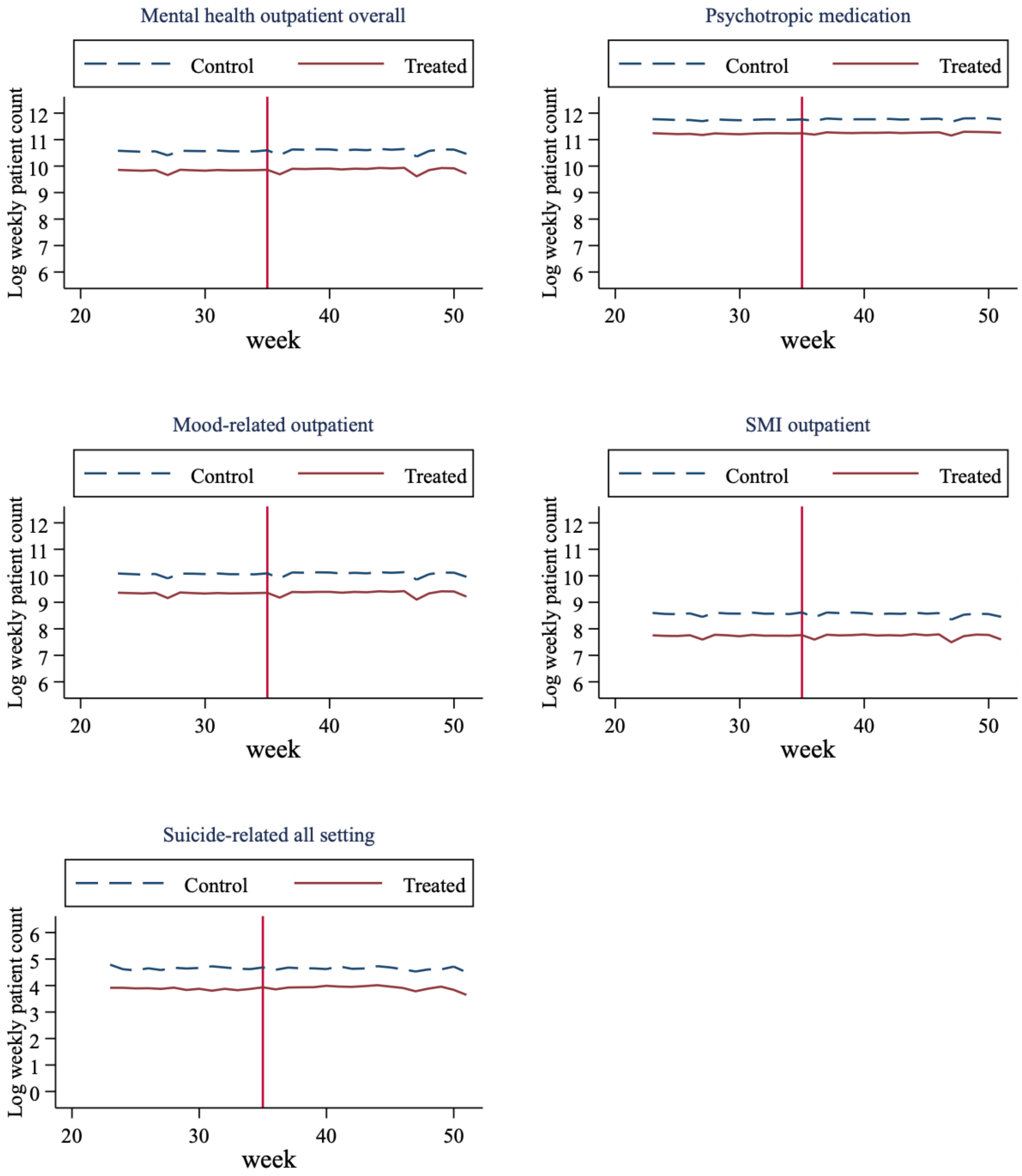

Notes: The trajectory plots are for Phase 2 analysis and include all five outcomes. The treated and synthetic trajectories are generally stable prior to treatment and do not exhibit obvious divergent trends before treatment exposure. Because the estimated effects for our statistically significant outcomes are small, we would not expect to see a clear visual separation in these plots.

© 2026 Ge Y et al. *JAMA Health Forum*.

eFigure 3. Event Study Plot Phase 1

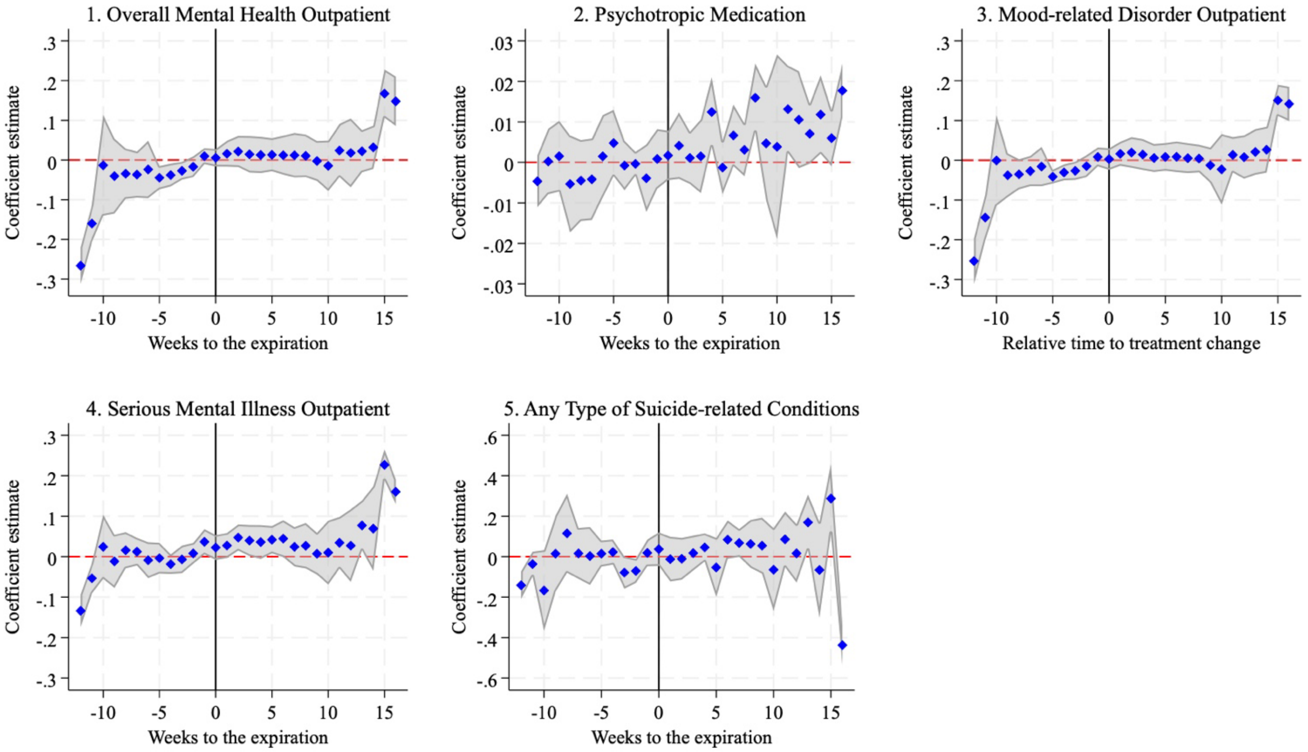

Notes: They are generated using the sdid_event package in Stata, which implements the event-study version of the synthetic difference-in-differences (SDID) estimator. These figures display the estimated dynamic SDID treatment effects relative to a baseline period. They are useful for visualizing the timing of estimated post-treatment effects, but they are not designed to serve as diagnostic plots of pre-treatment balance in outcome levels. A few panels show one or two early lead estimates that are more negative before moving back toward zero; we do not interpret these early isolated points as evidence of systematic pre-trends. In general, the lead estimates are imprecise and include zero within their confidence intervals. For the psychotropic medication and SMI outpatient outcomes, the post-treatment estimates are generally positive, and their confidence intervals are more often centered above zero.

© 2026 Ge Y et al. *JAMA Health Forum*.

eFigure 4. Event Study Plot Phase 2

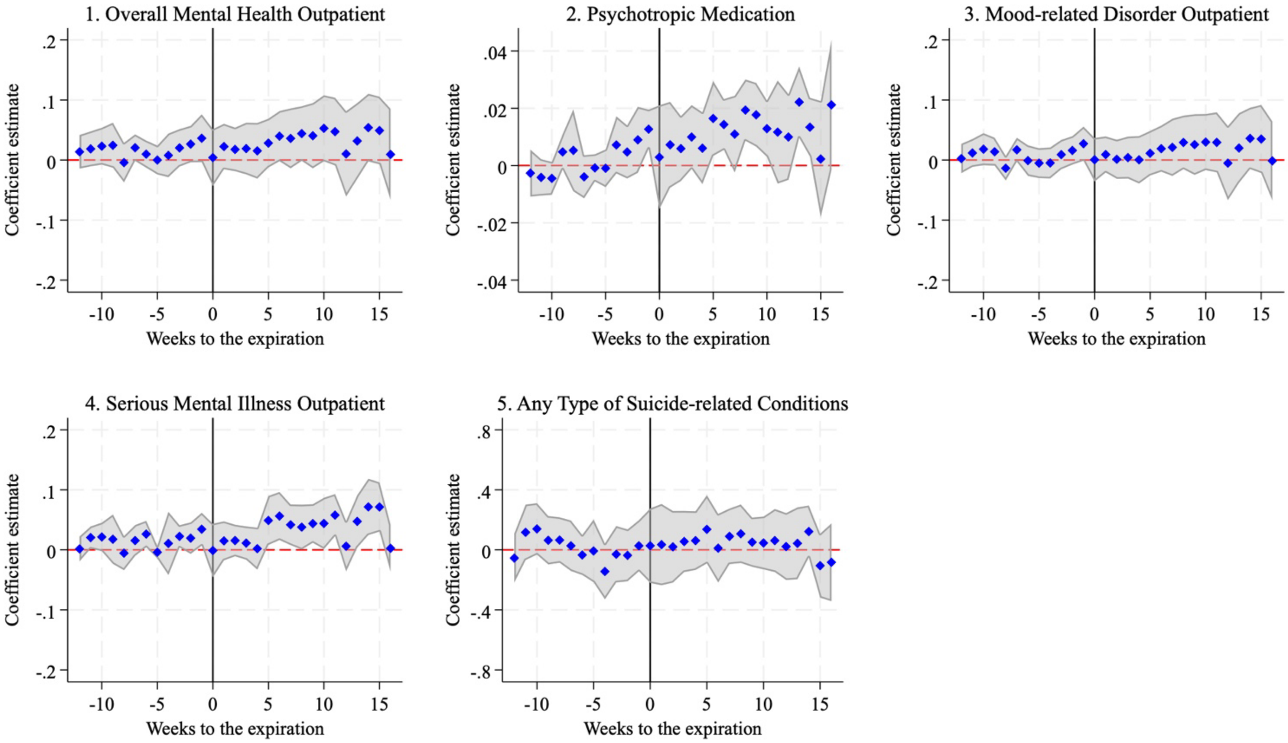

Notes: They are generated using the sdid_event package in Stata, which implements the event-study version of the synthetic difference-in-differences (SDID) estimator. These figures display the estimated dynamic SDID treatment effects relative to a baseline period. They are useful for visualizing the timing of estimated post-treatment effects, but they are not designed to serve as diagnostic plots of pre-treatment balance in outcome levels. In general, the lead estimates are imprecise and include zero within their confidence intervals. For the psychotropic medication and SMI outpatient outcomes, the post-treatment estimates are generally positive, and their confidence intervals are more often centered above zero.

© 2026 Ge Y et al. *JAMA Health Forum*.
