# Supplementary material for: Mental Health Care Utilization Following Eviction Moratorium Expirations
Source: JAMA Health Forum. 2026 May 22;7(5):e261212. doi: 10.1001/jamahealthforum.2026.1212 (PMC13197875; doi:10.1001/jamahealthforum.2026.1212)
Supplement: Supplement 2. — Data sharing statement [file jamahealthforum-e261212-s002.pdf]

Data Sharing Statement

Ge. Mental Health Care Utilization Following Eviction Moratorium Expirations. *JAMA Health Forum*. Published May 22, 2026. doi:10.1001/jamahealthforum.2026.1212

Data
Data available: No
